# Investigation of Active Anti-Inflammatory Constituents of Essential Oil from *Pinus koraiensis* (Sieb. et Zucc.) Wood in LPS-Stimulated RBL-2H3 Cells

**DOI:** 10.3390/biom11060817

**Published:** 2021-05-31

**Authors:** Jiyoon Yang, Won-Sil Choi, Ki-Joong Kim, Chang-Deuk Eom, Mi-Jin Park

**Affiliations:** 1 Division of Forest Industrial Materials, Department of Forest Products and Industry, National Institute of Forest Science, Seoul 02455, Korea; dldh89@korea.kr; 2 Division of Life Sciences, School of Life Sciences, Korea University, Seoul 02841, Korea; kimkj@korea.ac.kr; 3 National Instrumentation Center for Environmental Management, Seoul National University, Seoul 08826, Korea; choialla@snu.ac.kr; 4 Division of Wood Industry, Department of Forest Products and Industry, National Institute of Forest Science, Seoul 02455, Korea; willyeom@korea.kr

**Keywords:** *Pinus koraiensis*, essential oil, anti-inflammatory, active compound, *α*-pinene, *β*-pinene, *α*-terpineol, longifolene

## Abstract

In a previous study, we demonstrated the anti-inflammatory activity of the essential oil extracted from Korean pine (*Pinus koraiensis*, Sieb. et Zucc.) wood. This study aims to investigate the active anti-inflammatory constituents of *P. koraiensis* oil. The essential oil was extracted from *P. koraiensis* wood by hydrodistillation and was divided into six fractions (A–F) through fractional distillation. Then, the anti-inflammatory activities of the fractions (A–F) were determined. Fractions A and F markedly downregulated the production of pro-inflammatory cytokines as well as the secretion of *β*-hexosaminidase in lipopolysaccharide (LPS)-stimulated RBL-2H3 cells. The main constituents of the active anti-inflammatory A and F fractions were (+)-*α*-pinene, (−)-*β*-pinene, (+)-*α*-terpineol, 3-carene, (+)-limonene, and longifolene. These six single compounds decreased the expression of inflammatory-related genes (i.e., IL-4 and IL-13) as well as the secretion of *β*-hexosaminidase in LPS-stimulated RBL-2H3 cells. (+)-*α*-Pinene, (−)-*β*-pinene, (+)-*α*-terpineol, and longifolene exhibited the strongest effects; these effects were comparable to those of the positive control (i.e., dexamethasone). The findings indicate that the interactions between these components exhibit potential for the management and/or treatment of inflammatory conditions as well as base structures for the development of novel anti-inflammatory drugs.

## 1. Introduction

The Korean pine (*Pinus koraiensis* Siebold and Zuccarini) grows in Russia, Korea, China, and Japan at locations higher than 1000 m above sea level. It can reach 1 m in diameter and 20–30 m in height [1]. The lignum of *P. koraiensis* is hard and has been widely used in North Asia and Korea as a furniture material for a long time. Further, the seeds of *P. koraiensis* contain high-quality fatty acids, amino acids, carbohydrates, and vitamins [2]. As a result, *P. koraiensis* has been used as a food supplement and in traditional Asian medicine for thousands of years. Studies have reported that the extract of *P. koraiensis* bark exhibits antitumor, antioxidant, antiaging, and antimutagenic activities. Therefore, *P. koraiensis* is a species with very high economic value and has been selected as one of the 10 most economically and culturally important trees in Korea [3,4]. In addition, research has recently been conducted in various fields, including breeding of *P. koraiensis* and its use as a genetic resource [5].

Inflammation is a physiological response to a variety of agents, including infectious microorganisms, toxic chemicals, and physical injury [6]. Many diseases are associated with inflammation, including skin inflammation and auto-immune diseases, such as arthritis and diabetes, Alzheimer’s disease, and cancer [7].

Inflammation is mediated by a complex interaction of pro- and anti-inflammatory mediators, the perturbation of which is associated with an exacerbated release of pro-inflammatory cytokines and consequent deleterious effects [8]

Inflammatory mediators produced from activated mast cells and T helper type 2 (Th2) cells drive allergic inflammation. Th2 cells induce allergic inflammation through the production of cytokines, such as interleukin (IL)-4, IL-10, and IL-13 [9]. IL-4 and IL-13 are central to type-2 inflammation; therefore, they represent targetable candidates for the amelioration of allergic disease [10]. For example, IL-4 and IL-13 are required to drive most of the key processes associated with type-2 inflammation, including immunoglobulin E (IgE) production, mucus production, and innate cell recruitment to sites of inflammation [11,12]. Given the key role of IL-4 and IL-13 in type-2 inflammation, anti-inflammatory activity can be confirmed via changes in the expression levels of these two cytokines.

Many drugs that prevent or minimize the progression of inflammation are available, including non-steroidal anti-inflammatory drugs and corticosteroids. However, regular use of non-steroidal anti-inflammatory drugs results in a number of side effects, some of which can be very serious. Moreover, the prolonged use of corticosteroids can suppress pituitary–adrenal function, evoke hyperglycemia, and increase susceptibility to infections [13]. As a result, the use of natural medicine for the amelioration of inflammation has become an active research area.

The biological activities of many plants have been exploited in ethnomedicine for the treatment of inflammatory diseases. These biological properties are often imparted by essential oils contained in medicinal plants. Such essential oils exhibit different biological activities, including anti-inflammatory, antioxidant, antibacterial, antiaging, and anti-melanogenic activities [14,15].

In a previous study, we highlighted the anti-inflammatory effect of the essential oil (10^−7^%) extracted from *P. koraiensis* wood on lipopolysaccharide (LPS)-stimulated rat basophilic leukemia (RBL)-2H3 cells [16]. We assessed the effect of wood oil on IL-4 and IL-13 mRNA production using the LPS-stimulated RBL-2H3 cell line. The essential oil extracted from *P. koraiensis* wood significantly decreased the relative mRNA expression of IL-4 (to 36.8%) and IL-13 (to 42.9%) at 10^−7^%, compared with the LPS-stimulated RBL-2H3 cells. The *P. koraiensis* oil showed an inhibitory effect on the *β*-hexosaminidase release (to 72.6%), but the effect was insignificant compared with other oils. These results suggest that the essential oil had immunostimulatory effects on T cells and meaningfully inhibited allergy-associated cytokines IL-4 and IL-13. Although the anti-inflammatory effect of the essential oil extracted from *P. koraiensis* wood has been confirmed, its anti-inflammatory constituents have not been identified. Accordingly, the present work aims to study the bioactive constituents of the essential oil from *P. koraiensis* wood in LPS-stimulated RBL-2H3 cells.

## 2. Materials and Methods

### 2.1. Chemicals

(+)-*α*-Pinene (Cat #. 80604, purity ≥97.0%), (+)-*α*-terpineol (Cat #. 83073, purity ≥97.0%), 3-carene (Cat #. 94415-5ML, purity ≥97.0%), and *R*-(+)-limonene (Cat #. 62118-5ML, purity ≥95.0%) were purchased from Fluka (Buchs, Switzerland). (−)-*β*-Pinene (Cat #. 402753-10G, purity ≥97.0%) was purchased from Sigma-Aldrich (St. Louis, MO, USA). (+)-Longifolene (Cat #. 5395 S, purity ≥95.0%) was purchased from Extrasynthese (Rue Jacquard, Genay, France).

### 2.2. Plant Material

The wood of *P. koraiensis* (Sieb. et Zucc.) was collected from Gapyeong, Gyeonggi Province of Korea, in January 2015. The wood was milled and the sawdust obtained was used for the extraction of essential oil.

### 2.3. Extraction of Essential Oil and Its Fractioanl Distillation

The obtained sawdust of the *P. koraiensis* wood was hydrodistilled at atmospheric pressure using a Clevenger-type apparatus. A 10 L round-bottom flask containing 1.0 kg of sawdust was placed on a digital heating mantle (MTOPS) and 6.0 L of distilled water was poured into the flask, which was then connected to the Clevenger-type apparatus. The sawdust was extracted for 7 h. The obtained glass green (Pantone color. 11-0205 TPG) essential oil was dried over anhydrous sodium sulfate (Samchun) and filtered through a 0.45 µm membrane disk filter (ADVANTEC). For the separation of essential oil, fractional distillation was performed under atmospheric pressure [17].

The oil fractions (A, B, C, D, E and F) obtained at different temperatures are presented in Table 1.

The six oil fractions were then transferred to dark vials and stored at 4 °C until further analysis. The yield of the oil fractions was calculated using the following equation (Equation (1)):

(1) $$Oil\;fraction\;yield\;\left(\%\right)=\frac{Recovered\;mass\;of\;the\;essential\;oil\;\left(g\right)}{Initial\;mass\;of\;the\;essential\;oil\;\left(g\right)}\times 100$$

### 2.4. GC–MS Analysis

To identify the volatile components in the oil fractions, the six oil fractions (A–F) were analyzed by GC–MS (Thermo Fisher Scientific, Waltham, MA, USA) using the DB-5MS capillary column (30 m × 0.25 mm; 0.25 μm; Thermo Scientific, Waltham, MA, USA) and a flame ionization detector (FID) as described previously (Table 2) [16].

The components of the six oil fractions were identified based on peaks with the highest spectral matching using the National Institute of Standards and Technology (NIST; Gaithersburg, MD, USA) library search program (version 11). The Kovats retention index (KI) values of the individual compounds were determined by comparing their relative retention times with those of an *n*-alkane mixture (C_8_–C_20_, Sigma-Aldrich, Missouri, USA) using the DB-5MS column (Thermo Scientific). The essential oil components were identified by comparing their calculated KI values with literature values (e.g., NIST Chemistry WebBook).

### 2.5. Cell Culture

The rat basophilic leukemia cell line (RBL-2H3, KCLB no. 22256) was obtained from Korea Cell Line Bank (Seoul, Korea). RBL-2H3 cells were cultured in Dulbecco’s modified Eagle’s medium (DMEM, Welgene, Gyeongsan-si, Korea) supplemented with 10% fetal bovine serum (FBS, Gibco, Co Dublin, Ireland), 1% penicillin–streptomycin (Gibco), and 0.4 μL/mL Plasmocin (Invivogen, San Diego, CA, USA) at 37 °C in a 5% CO_2_ incubator (Panasonic, Osaka, Japan).

### 2.6. Cell Cytotoxicity

The microdilution method of CLSI M07-49 was modified and used to evaluate the cell cytotoxicity (10^−5^–10^−7^%) [18]. Cell cytotoxicity was measured using Cell Counting Kit (CCK)-8 (DoGenBio, Seoul, Korea) according to the manufacturer’s instructions. Briefly, the oil fractions and single compounds were prepared in DMSO. A stock solution of the ingredients to be tested were prepared by diluting 100 μL ingredients in 900 μL DMSO. The cells were seeded into 96-well plates at a density of 8 × 10^3^ cells/well, and then, stock solutions were added to the well plate to give a final concentration of 10^−5^–10^−7^% for 24 h. After treatment, 10 µL of CCK-8 solution was added to each well and the 96-well plate was incubated at 37 °C for 1 h. Then, the optical density (OD) value of each well was determined using a microplate reader (Epoch, Winooski, VT, USA) at a wavelength of 450 nm to obtain the cell viability data. The percentage of viable cells was calculated using the following equation (Equation (2)):

(2) $$Cell\;viability\left(\%\right)=\frac{\left(At-Ab\right)}{\left(Ac-Ab\right)}\times 100$$

where, *At* is the absorbance of treated cells, *Ab* is the absorbance of the blank, and *Ac* is the absorbance of the control.

### 2.7. Determination of the Levels of Cytokines IL-4 and IL-13 via Quantitative Real-Time PCR

Total RNA was isolated from RBL-2H3 cells as done in a previous study [19]. RBL-2H3 cells were grown on 6-well plates (3 × 10^5^ cell/well) for 24 h. The cells were stimulated with 1 μg/mL LPS (Sigma-Aldrich) for 1 h and then washed twice with DPBS (Gibco). The cells were treated with 200 μL dexamethasone, oil fraction (A–F), and the single compounds (10^−7^%) for 24 h. Total RNA was isolated using the TRIzol reagent (Invitrogen) and quantified using a spectrophotometric microplate reader at 260 and 280 nm. Total RNA was reverse transcribed into first-strand complementary DNA (cDNA) using M-MLV reverse transcriptase (Invitrogen) and random primers (Takara Bio Inc., Shiga, Japan). Each cDNA sample was amplified with 2× SYBR^®^ Premix Ex Taq (Takara Bio Inc.) and 3 pmol of each primer. The oligonucleotide primers used for PCR (Foster) amplification that were obtained from target cellular RNA are listed in Table 3.

PCR amplification was performed under 40 cycles of denaturation at 95 °C for 15 s, annealing at 60 °C for 30 s, extension at 72 °C for 30 s, and final elongation at 72 °C for 10 min. The IL-4 and IL-13 gene expression C_t_ values were normalized to the corresponding values for the 18S gene expression using the RQ software (version 1.3, Applied Biosystems, Bedford, MA, USA).

### 2.8. β-Hexosaminidase Secretion Assay

The degranulation response of RBL-2H3 cells was quantified by measuring the level of *β*-hexosaminidase released in the supernatants [20]. RBL-2H3 cells were grown on 6-well plates at a density of 2 × 10^5^ cells for 24 h. The cells were then treated with 800 ng/mL DNP-specific IgE (Sigma-Aldrich) overnight. To remove the excess IgE, the cells were washed twice with Tyrodes’ assay buffer (pH 7.3). The cells were then stimulated with DNP-BSA (Invitrogen), suspended in 500 µL of extracellular buffer with 0.1% BSA, and incubated at 37 °C for 1 h. After incubation, 50 µL of the supernatant was incubated with 200 µL of 1 mM *p*-nitrophenyl-*N*-acetyl-*β*-D-glucosaminide (Sigma-Aldrich) in 0.05 M citrate buffer (pH 4.5) at 37 °C for 1 h. The enzyme reaction was terminated by adding 500 µL of sodium carbonate buffer. The OD of each reaction was recorded at 405 nm. The percentage of viable cells was calculated using the following equation (Equation (3)):

(3) $$\beta -\mathrm{hexosaminidase}\;\sec\mathrm{retion}\;\left(\%\right)=\frac{Am}{\left(Al-Am\right)}\times 100$$

where, *Al* is the absorbance of the cell lysate and *Am* is the absorbance of the medium.

### 2.9. Statistical Analysis

The results are expressed as mean ± standard deviation (*n* = 3). Differences between vehicle control and treatment groups were tested using two-way ANOVA followed by multiple comparisons by the Tukey test. The *p* values lower than 0.05 were considered statistically significant.

## 3. Results

### 3.1. Yield and Chemical Composition

*P. koraiensis* wood oil was divided into six fractions (A–F) using fractional distillation. The yields of the fractions are presented in Table 4.

The chemical compositions of the oil fractions as determined via GC–MS are presented in Table 5.

**Figure 1:**
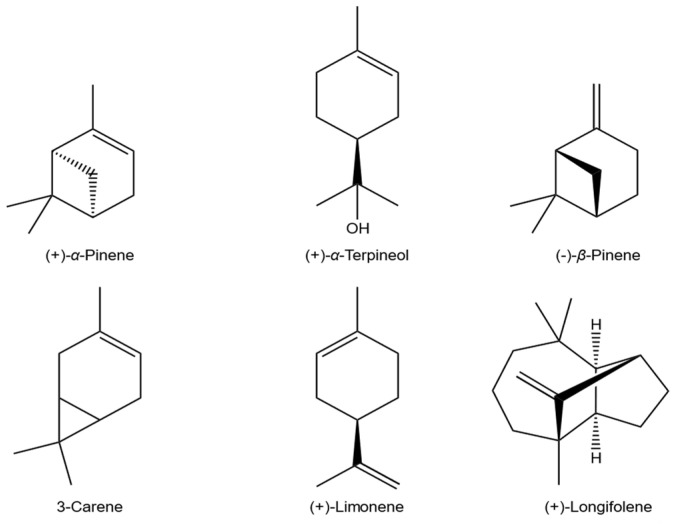
Chemical structures of the main components in the six oil fractions.

As shown in Table 5, the main components of fractions A, B, C, and D were *α*-pinene, *β*-pinene, 3-carene, and limonene. Furthermore, the contents of monoterpenes in fractions A, B, C, and D were higher than those of sesquiterpenes. By contrast, the major constituents of fraction E were *α*-terpineol (16.06%) and limonene (12.82%). Longifolene (27.27%) and α-terpineol (16.61%) were the predominant components in fraction F (Figure 1).

### 3.2. Cell Cytotoxicity

**Figure 2:**
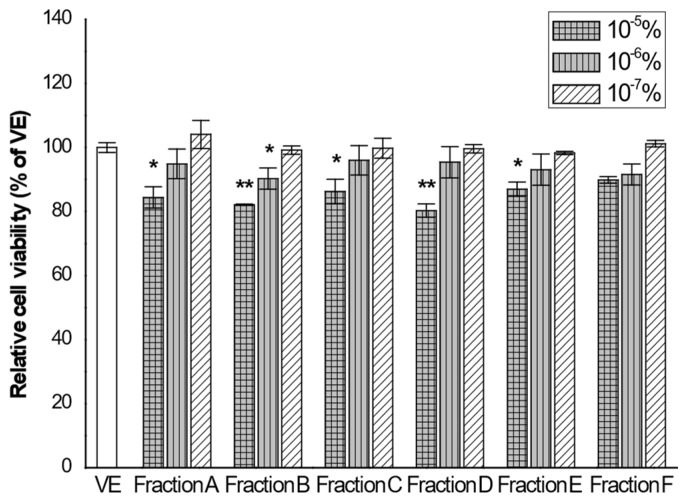
Effects of the six oil fractions on RBL-2H3 cell viability. RBL-2H3 cells were seeded in 96-well plate and treated with a series of concentration of the six oil fractions (10^−5^, 10^−6^, and 10^−7^%) for 24 h. Cell viability was assessed by cell counting kit-8 assay. VE: vehicle, DMSO. The results are presented as mean ± standard deviations. * *p* < 0.05 and ** *p* < 0.01 compared to VE.

The CCK-8 assay was used to determine the cytotoxicity of the oil fractions against RBL-2H3 cells. The results of cell cytotoxicity are shown in Figure 2. The individual oil fractions exhibited toxicity in a dose-dependent manner. We found that no sample exhibited a general cytotoxicity, defined as a cell viability below 80%, as compared with vehicle, in the RBL-2H3 cells [21].

Fraction A exhibited an IC_50_ value of 0.063%, whereas IC_50_ values of 0.113%, 0.121%, and 0.06% were recorded for fractions B, C, and D, respectively. IC_50_ values of fractions E and F were >10^−4^%.

Therefore, to evaluate the anti-inflammatory effects of the oil fractions, RBL-2H3 cells were treated with oil fractions at a concentration of 10^−7^%.

### 3.3. Effects of the Oil Fractions on the Expression of IL-4 and IL-13

**Figure 3:**
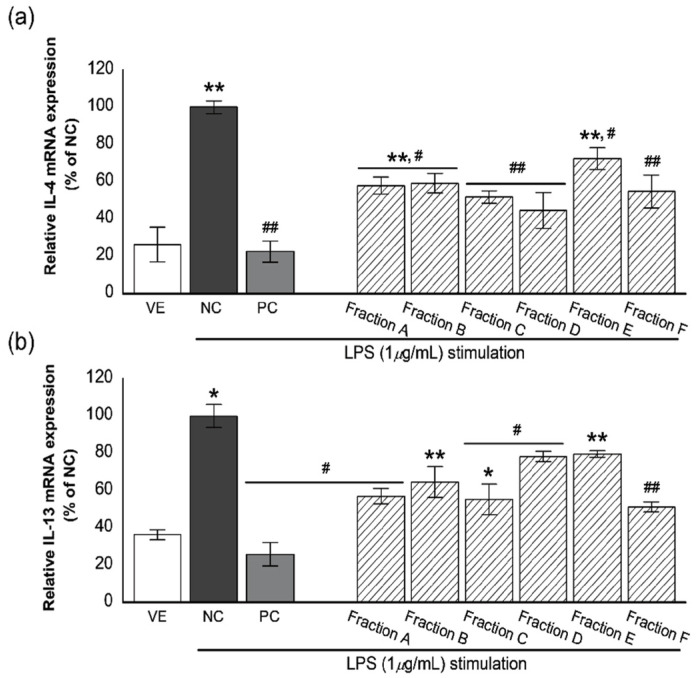
Effect of the six oil fractions on (**a**) IL-4 and (**b**) IL-13 mRNA expression levels in LPS-stimulated RBL-2H3 cells. VE: vehicle, DMSO; NC: negative control, 1-μ*g*/mL LPS treated group; PC: positive control, 100-nM dexamethasone treated group, an oil fraction (A–F) (10^−7^%). Values are presented as mean ± standard deviation. * *p* < 0.05 and ** *p* < 0.01 compared with VE; ^#^ *p* < 0.05, ^##^ *p* < 0.01 compared with NC.

The modulation of inflammatory cytokines from mast cells is a key indicator of reduced allergic symptoms. In particular, the regulation of IL-4 and IL-13 is considered the most important therapeutic strategy for allergic inflammatory conditions [22]. Therefore, we determined whether the oil fractions from *P. koraiensis* wood oil regulate the levels of proinflammatory cytokines IL-4 and IL-13 in RBL-2H3 cells (Figure 3).

Stimulation of RBL-2H3 cells with LPS significantly increased the relative expression level of IL-4 (Figure 3a). However, this increase in IL-4 expression was suppressed in LPS-stimulated RBL-2H3 cells treated with dexamethasone (about 77.5% reduction), a steroidal inflammatory drug. Anti-inflammatory activities of fractions at a 10^−7^% concentration showed 27.6–55.5% inhibition compared with negative control. Inhibition of IL-4 expression by fractions are as follows: 42.1%, 40.9%, 48.3%, 55.5, 27.6%, and 45.3% inhibition for fractions A–F, respectively. Fractions A, C, D, and F showed a higher inhibitory effect of IL-4 expression than other fractions. However, these fractions had a lower inhibitory effect than dexamethasone, positive control.

A remarkable increase in IL-13 expression compared with that of vehicle (VE) was also observed when RBL-2H3 cells were treated with LPS. However, this increase was significantly suppressed following treatment of the cells with dexamethasone and fractions (Figure 3b) as follows: 74.1% inhibition for dexamethasone and 43.0%, 35.4%, 44.7%, 21.8%, 20.4%, and 48.8% inhibition for fractions A–F, respectively. Fractions A, C, and F showed a higher inhibitory effect of IL-13 expression than other fractions.

### 3.4. Effects of the Oil Fractions on β-Hexosaminidase Release

**Figure 4:**
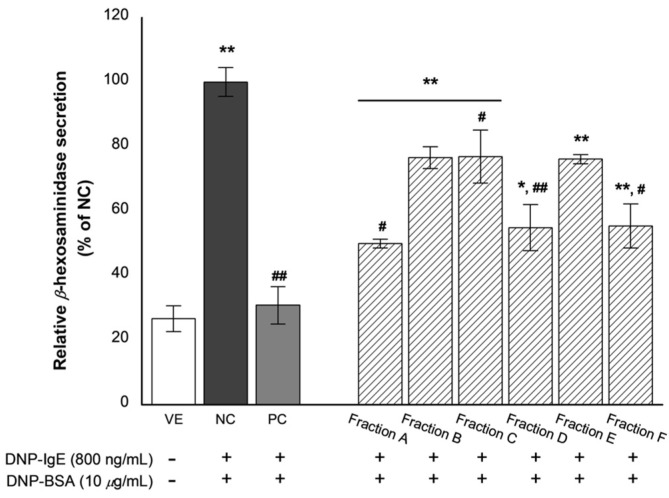
Inhibitory effect of the six oil fractions on *β*-hexosaminidase release. VE: vehicle, DMSO; NC: negative control, 800 ng/mL DNP-IgE and 10-μg/mL DNP-BSA treated group; PC: positive control, 100-nM dexamethasone treated group, an oil fraction (A–F) (10^−7^%). Values are presented as mean ± standard deviation. * *p* < 0.05 and ** *p* < 0.01 compared with VE; ^#^ *p* < 0.05, ^##^ *p* < 0.01 compared with NC.

*β*-Hexosaminidase is present in the granules of basophils or mast cells and is secreted by these cells upon allergic reactions. *β*-Hexosaminidase secretion can be used to indicate and quantify the extent of degranulation [23]. The effects of the oil fractions on *β*-hexosaminidase release are shown in Figure 4.

*β*-Hexosaminidase secretion in the negative control (NC) group, stimulated with DNP-BSA and with promoted degranulation, was approximately three times higher than that of the VE group. However, treatment of the inflammatory-induced RBL-2H3 cells with dexamethasone results in a significant decrease in the relative secretion of *β*-hexosaminidase compared with the NC (about 69.2% decrease). Similarly, *β*-hexosaminidase secretion was significantly reduced following treatment with the fractions. Subsequently, the oil fractions exhibited considerable anti-inflammatory activity by inhibiting *β*-hexosaminidase secretion in LPS-induced RBL-2H3 cells as follows: 50.1%, 23.4%, 23.1%, 45.1%, 23.9%, and 44.6% inhibition for fractions A–F, respectively. Of all the fractions, A, D, and F were the most potent.

### 3.5. Effects of Single Compounds on Cell Viability

**Figure 5:**
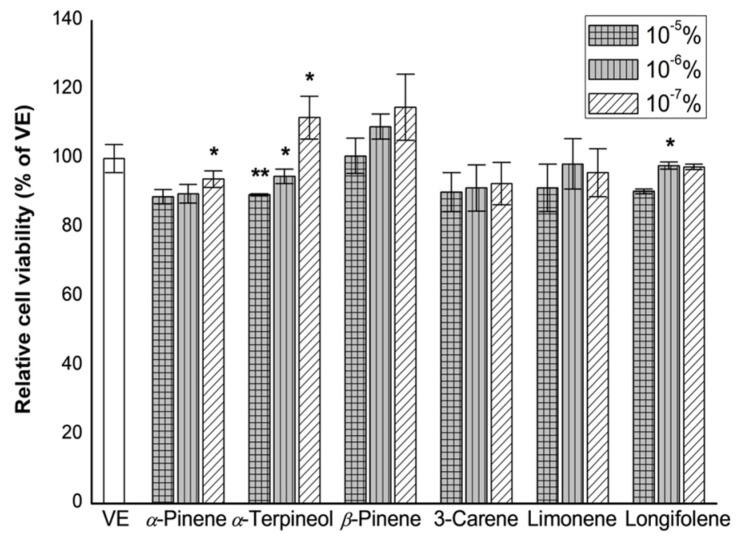
Effect of single compounds on the RBL-2H3 cell viability. RBL-2H3 cells were seeded in 96-well plate and treated with a series of concentration of the six single compounds (10^−5^, 10^−6^, and 10^−7^%) for 24 h. Cell viability was assessed by cell counting kit-8 assay. VE: vehicle, DMSO. The results are presented as mean ± standard deviation. * *p* < 0.05 and ** *p* < 0.01 compared with VE.

The anti-inflammatory effects of six standard substances [(+)-*α*-pinene, (−)-*β*-pinene, (+)-*α*-terpineol, 3-carene, limonene, longifolene], the main components of fractions A, D, and F, which have excellent anti-inflammatory activity, were investigated. In particular, to determine the effect of six standard substances on the survival and proliferation of RBL-2H3 cells, the CCK assay was performed by treating the cells with a concentration range of 10^−7^–10^−5^% (Figure 5).

Cell viability of six single compounds is shown in Figure 5. The six single compounds did not show any cytotoxicity at the investigated concentration in this study. Therefore, the anti-inflammatory effects of the six single compounds were evaluated at 10^−7^% concentration.

Both (+)-*α*-pinene and (−)-*β*-pinene exhibited an IC_50_ value of 0.033%. The IC_50_ value of (+)-*α*-terpineol was 0.030%. However, IC_50_ values of 0.021%, 0.021%, and 0.028% were recorded for 3-carnene, limonene, and longifolene, respectively.

### 3.6. Effects of Single Compounds on the Expression of IL-4 and IL-13

**Figure 6:**
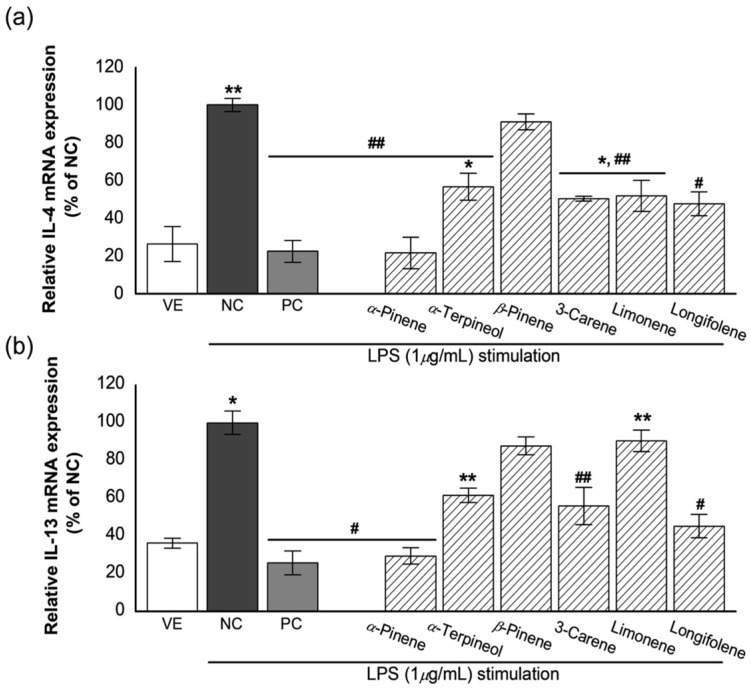
Effects of the single compounds on (**a**) IL-4 and (**b**) IL-13 mRNA expression in LPS-stimulated RBL-2H3 cells. VE: vehicle, DMSO; NC: negative control, 1-μg/mL LPS treated group; PC: positive control, 100-nM dexamethasone treated group, the single compounds (10^−7^%). Values are presented as mean ± standard deviation. * *p* < 0.05 and ** *p* < 0.01 compared with VE; ^#^ *p* < 0.05, ^##^ *p* < 0.01 compared with NC.

The inflammatory response induced RBL-2H3 cells were treated with the six single compounds. The results are shown in Figure 6.

As shown in Figure 6a, LPS treatment increased the relative expression level of IL-4 by >3.8 times compared with VE treatment. Upon treatment with dexamethasone (PC), the IL-4 gene expression level in NC was similar to that in VE (about 77.5% reduction). In addition, the relative IL-4 expression levels upon treatment with the six single compounds were decreased in VE compared with that in NC as follows: 78.4% inhibition for (+)-*α*-pinene, 9.0% inhibition for (−)-*β*-pinene, 43.3% inhibition for (+)-*α*-terpineol, 49.7% inhibition for 3-carnene, 48.2% inhibition for limonene, and 52.4% inhibition for longifolene. (+)-*α*-Pinene was found to be more effective than the other single compounds, followed by longifolene, 3-carnene, limonene, and (+)-*α*-terpineol.

Similar results were observed regarding the relative expression level of IL-13 (Figure 6b). The six single compounds exhibited anti-inflammatory activity compared with NC. Inhibitions of IL-13 expression by single compounds were as follows: 74.1% for dexamethasone, 70.6% for (+)-*α*-pinene, 38.4% for (+)-*α*-terpineol, 12.2% for (−)-*β*-pinene, 44.1% for 3-carnene, 9.5% for limonene, and 54.7% for longifolene against NC. Inhibition of IL-13 expression by (+)-*α*-pinene was more effective than the other compounds. The activity of (+)-α-pinene was similar to that of dexamethasone (PC). Longifolene and 3-carene also had an inhibitory effect on the IL-13 expression.

### 3.7. Effects of Active Compounds on β-Hexosaminidase Release

**Figure 7:**
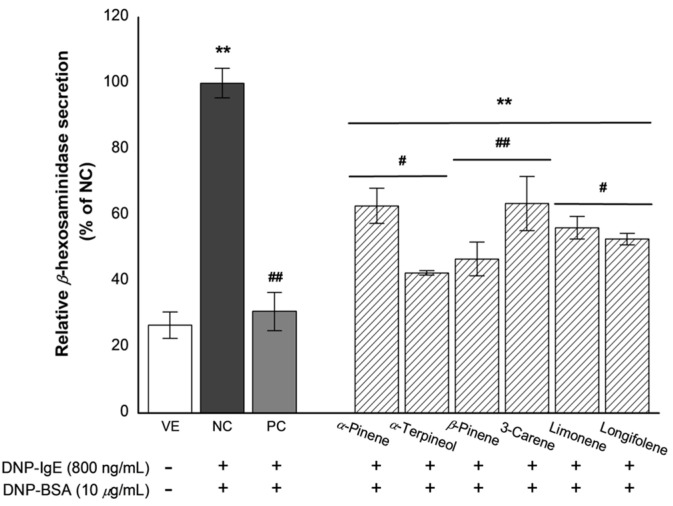
Inhibition effect of the single compounds on *β*-hexosaminidase release. VE: vehicle, DMSO; NC: negative control, 800-ng/mL DNP-IgE and 10-μ*g*/mL DNP-BSA treated group; PC: positive control, 100-nM dexamethasone treated group, the single compounds (10^−7^%). Values are presented as mean ± standard deviation. ** *p* < 0.01 compared with VE; ^#^ *p* < 0.05, ^##^ *p* < 0.01 compared with NC.

The effects of the six single compounds on *β*-hexosaminidase secretion are graphically illustrated in Figure 7.

The RBL-2H3 cells stimulated with DNP-BSA promoted degranulation and significantly increased *β*-hexosaminidase secretion (NC). The tested single compounds had inhibitory effects on *β*-hexosaminidase secretion from DNP-BSA-stimulated RBL-2H3 cells. Inhibition of *β*-hexosaminidase secretion by single compounds were as follows: 69.2% for dexamethasone, 37.2% for (+)-*α*-pinene, 57.5% for (+)-*α*-terpineol, 53.3% for (−)-*β*-pinene, 36.5% for 3-carnene, 43.8% for limonene, and 47.3% for longifolene against NC. Moreover, the inhibitory effects of (+)-*α*-terpineol and (−)-*β*-pinene were high. These results indicate that (+)-*α*-terpineol and (−)-*β*-pinene in the essential oil from *P. koraiensis* wood are the highest contributors of the inhibition in *β*-hexosaminidase secretion.

## 4. Discussion

Inflammatory diseases are generally treated with steroidal or non-steroidal anti-inflammatory drugs. However, both have significant negative side effects, thereby limiting their use in certain sections of the population [24]. As a result, the development of new drugs with novel modes of action and fewer side effects is warranted. Essential oils are secondary metabolites that are extensively used in aromatherapy and various traditional medicinal systems. Many of these oils possess different pharmacological properties.

Immune responses are regulated by a highly complex and intricate network of control elements. This reaction causes degranulation of mast cells and facilitates the release of primary chemical mediators such as histamine. At this time, cell membranes are activated and then secondary chemical mediators are secreted. Due to this, cytokines (i.e., IL-3, IL-4, and IL-5, etc.) are secreted from mast cells. Among these, the key regulatory components are anti-inflammatory cytokines and specific cytokine inhibitors [25]. IL-4 and IL-13 share a common cellular receptor (IL-4 type 1 receptor); this accounts for many of the similarities between the actions of these two anti-inflammatory cytokines [26]. The principal functional difference between IL-4 and IL-13 lies in their effect on T cells. *β*-Hexosaminidase is used as a typical marker of mast cell degranulation. Approximately 85% of *β*-hexosaminidase contained in mast cells is localized in the granules [27]. Based on this, *β*-hexosaminidase is commonly used as a biomarker of mast cell degranulation caused by various stimuli. Therefore, active anti-inflammatory constituents may be different depending on the target due to these differences in anti-inflammatory mechanisms [28].

The most well-known endotoxin for inducing an anti-inflammatory response in vitro is lipopolysaccharides (LPS). LPS stimulates monocytes/macrophages through TLR4, resulting in the activation of a series of signaling events that potentiate the production of inflammatory mediators [29]. However, LPS is a model that does not correctly reproduce the actual process [30]. Nevertheless, many studies have been used because it is a strong immunostimulant when tested in vitro. In this study, LPS was also used as an inflammatory stimulator to search for anti-inflammatory constituents.

The anti-inflammatory effect of essential oil from the wood of *P. koraiensis* was demonstrated in our previous study [16]. The present study investigates the active anti-inflammatory constituents in the essential oil. Fractional distillation was used to separate essential oil into several fractions. As shown in Table 5, the main chemical compositions of each fraction were found to be different. Early fractions obtained by fractional distillation mainly comprised monoterpenes, which typically have low boiling points. However, sesquiterpenes, which typically have high boiling points, were the main components of late fractions. According to the results of GC–MS analysis, the main components of fractions A, B, C, and D were *α*-pinene (22.78–58.26%), *β*-pinene (13.19–29.67%), 3-carene (2.49–11.67%), and limonene (1.93–14.54%). By contrast, the major constituents of fraction E and F were *α*-terpineol (16.69, 16.98%) and longifolene (2.61, 27.87%).

To investigate active anti-inflammatory constituents of *P. koraiensis* oils, the effects of fractions on the expression of inflammation-related genes (i.e., IL-4 and IL-13) and secretion of *β*-hexosaminidase were examined. As mentioned earlier, IL-4 and IL-13 have many similarities in the inflammatory response. Fractions that effectively inhibit IL-4 and IL-13 expression were the same as A, C, and F. There was no statistical significance detected in the expression of inflammation-related genes (i.e., IL-4 and IL-13) between fractions. In contrast, a result of *β*-hexosaminidase secretion showed a different trend. In the inhibition of *β*-hexosaminidase secretion, fraction A, D, and F were more active than other fractions. In conclusion, the fractions that most effectively regulated the anti-inflammatory cytokines and the secretion of *β*-hexosaminidase were A and F. As a result of GC-MS, the main constituents of fraction A were (+)-*α*-pinene (58.26%) and *β*-pinene (13.19%). Additionally, the main components of fraction F were *α*-terpineol (16.98%) and longifolene (27.87%).

The anti-inflammatory activity with the major components of each fraction was evaluated. The gene expression level of IL-4 and IL-13 were reduced significantly by six single compounds used in this study. In particular, (+)-*α*-pinene among the six compounds, effectively suppressed the gene expression of both IL-4 and IL-13. (+)-*α*-Pinene and (−)-*β*-pinene showed statistical significance in IL-4 and IL-13 gene expression (*p* = 0.001; *p* = 0.048). In addition, (+)-*α*-pinene and limonene showed a statistical significance of *p* = 0.034 in IL-13 gene expression. Other single compounds showed no statistical significance. From the results, although six single compounds had anti-inflammatory activities, (+)-*α*-pinene had the best inhibitory effects on IL-4 and IL-13 gene expression. Additionally, it was found that inhibitory effects of *α*-terpineol, 3-carene, and longifolene on IL-4 and IL-13 gene expression were similar. All six single compounds also had an inhibitory effect on the release of *β*-hexosaminidase. There was no statistical significance between the single compounds. The single compounds that suppressed *β*-hexosaminidase secretion very efficiently were (−)-*β*-pinene and (+)-*α*-terpineol. Based on these results, it is suggested that six single compounds had anti-inflammatory activities.

Fractions A and C, active anti-inflammatory fractions, were mainly consisted of (+)-*α*-pinene, (−)-*β*-pinene, (+)-*α*-terpineol, 3-carene, and limonene, which had anti-inflammatory activities. Faction A and C contained these constituents in high portions of 76.14% and 87.7%, respectively. For this reason, fractions A and C exhibited positive activities in gene expression of IL-4 and IL-13.

The major constituents of fraction F were *α*-terpineol (16.98%) and longifolene (27.87%). Although active anti-inflammatory constituents such as (+)-*α*-pinene, (−)-*β*-pinene, 3-carene were not contained in fraction F, fraction F had inhibitory effects on gene expression of IL-4, IL-13 and *β*-hexosaminidase secretion.

Fraction E contained *α*-terpineol (16.69%) and longifolene (2.61%) like fraction F but had less anti-inflammatory activity than fraction F. These results seem to be due to the difference in the portion of major constituents in each fraction. The proportions of (+)-*α*-terpineol in fractions E and F were similar, but the proportions of longifolene in F fraction was higher than fraction E. It was accordingly inferred that longifolene showed a significant effect on anti-inflammatory activity. Additionally, longifolene was expected to be an active anti-inflammatory compound.

In the present study, (+)-*α*-terpineol and *β*-pinene were identified as the potent constituent of the essential oil against *β*-hexosaminidase secretion. Previous studies have revealed the various biological properties of (+)-*α*-terpineol and *β*-pinene. (+)-*α*-Terpineol exerts a wide range of biological actions in humans, animals, and plants [31,32]. Further, it possesses a wide range of biological applications as anti-hypertensive, antiproliferative effects, and potent inhibitor of superoxide production [33]. For instance, it has been identified as one of the most important bioactive constituents of many essential oils. *β*-pinene displayed antimicrobial, anti-tumor, anti-inflammatory, antioxidant, and analgesic effects [34]. According to the results of this study, (−)-*β*-pinene activity was not prominent. In previous studies, there was a substantial difference in the activity of the two enantiomers; the two isomers behaved differently in vitro. In a chiral environment, one enantiomer may display different chemical and pharmacologic behavior than the other enantiomer [35]. Consequently, it is necessary to evaluate the anti-inflammatory activity of (+)-*β*-pinene, the enantiomer of (−)-*β*-pinene.

Taken together, the results presented in this study suggest the applications of essential oils or their components as anti-inflammatory agents and might accelerate the development of new drugs for different inflammatory diseases. Essential oils could be exploited as effective alternatives or complementary compounds of the chemical industry without inducing the same secondary effects.

## 5. Conclusions

Although several previous studies have demonstrated the anti-inflammatory effects of *P*. *koraiensis* wood oil, the active anti-inflammatory constituents of the oil have not been identified. Therefore, this study explored the in vitro anti-inflammatory effects of the fractions obtained from *P. koraiensis* wood oil as a means to investigate the anti-inflammatory constituents in the essential oil. GC–MS analysis revealed that the chemical compositions of the six oil fractions were different and identified the six most effective single components. The six single compounds were found to exhibit anti-inflammatory activities; among them, (+)-*α*-pinene, (−)-*β*-pinene, *α*-terpineol, and longifolene showed the highest activity. Therefore, four single compounds were assumed to be the most contributing compounds to the anti-inflammatory activity of *P. koraiensis* wood oil. Therefore, we decided that (+)-*α*-pinene, (−)-*β*-pinene, *α*-terpineol, and longifolene have a high contribution to anti-inflammatory activity in essential oil from *P. koraiensis* wood. Additionally, the minor components are critical to the synergistic activity [36].

Thus, (+)-*α*-pinene, (−)-*β*-pinene, *α*-terpineol, and longifolene may be useful for the prevention and treatment of inflammatory diseases. Furthermore, our findings provide a scientific rationale for the future utilization of *P. koraiensis*, (+)-*α*-pinene, (−)-*β*-pinene, *α*-terpineol, and longifolene.

## Figures and Tables

**Table 1:** Temperature conditions for the fractionation of the essential oil of *P. koraiensis* wood.

Oil Fraction	Temperature Range (°C)
A	50–65
B	65–75
C	75–81
D	81–82
E	82–94
F	Residues

**Table 2:** Operating parameters of GC–MS.

Column	DB-5MS capillary column		
	(30 m × 0.25 mm; 0.25 μm; Thermo Scientific, USA)		
GC oven conditions	50 °C, hold 5 min		
	10 °C/min to 65 °C, hold 30 min		
	5 °C/min to 120 °C, hold 10 min		
	5 °C/min to 180 °C		
	5 °C/min to 210 °C, hold 10 min		
	20 °C/min to 325 °C, hold 10 min		
Carrier gas	He (1 mL/min, 25 psi)		
Linear velocity	19.8 cm/s		
Injection mode	Split 1:20		
Injection temperature	250 °C		
MS parameters		FID parameters	
MS ionization mode	EI	FID temperature	300 °C
Scan time	0.2 s	Hydrogen flow	35.0 mL/min
Mass range	35–550 amu	Air flow	350.0 mL/min
Ion source temperature	270 °C	Make up flow	40.0 mL/min
Interface temperature	250 °C		

**Table 3:** Oligonucleotide primer sequences used for quantitative real-time polymerase chain reaction.

Gene	Primer Sequence (5′–3′)	Accession No.
IL-4	F: TGA TGT ACC TCC GTG CTT GA R: AGG ACA TGG AAG TGC AGG AC	X16058
IL-13	F: CTG GAA TCC CTG ACC AAC AT R: CCA TAG CGG AAA AGT TGC TT	L26913
GAPDH	F: CCA CAG TCC ATG CCA TCA C R: TCC ACC ACC CTG TTG CTG TA	NM_017008.4

F; forward, R; reverse.

**Table 4:** Yields of the fractions (A–F) isolated from *P. koraiensis* wood oil.

Oil	Weight of Fraction (g)	Yield (%)
Fraction A	4.0	15.2
Fraction B	0.7	2.6
Fraction C	6.1	23.4
Fraction D	1.4	5.5
Fraction E	1.7	6.4
Fraction F	12.3	46.9

**Table 5:** Chemical compositions of six fractions identified by GC–MS analysis.

KI ^a^	Compound Name ^b^	Area %						
		Wood Oil	Fraction A	Fraction B	Fraction C	Fraction D	Fraction E	Fraction F
Monoterpene hydrocarbons								
913	*β*-Ocimene	-	-	-	-	0.01	-	-
919	Tricyclene	0.08	0.23	0.24	0.17	0.05	-	-
921	*α*-Thujene	-	0.11	0.12	0.09	-	-	-
928	(+)-*α*-Pinene	27.00	58.26	62.99	54.66	22.78	3.34	-
935	(−)-*β*-Citronellene	-	0.08	0.09	0.08	0.04	-	-
941	(+)-Camphene	0.14	0.25	0.29	0.29	0.20	0.05	-
943	(−)-Camphene	1.10	1.93	2.27	2.40	1.80	0.45	-
946	2,4(10)-Thujadien	0.13	0.17	0.20	0.18	0.10	0.07	-
973	(−)-*β*-Pinene	11.15	13.19	16.54	21.95	29.67	6.65	-
986	*β*-Myrcene	0.23	0.15	0.21	0.26	0.27	0.06	-
1013	*α*-Phellandrene	-	0.03	0.05	0.08	0.16	0.11	-
1017	3-Carene	3.05	2.49	3.32	5.44	11.67	4.30	-
1030	*α*-Terpinene	0.33	0.17	0.27	0.48	1.05	0.68	-
1040	Cymene	1.32	0.77	1.09	1.91	5.71	6.66	-
1045	(+)-Limonene	3.47	1.93	2.68	4.92	14.54	13.32	-
1047	Sabinene	0.16	0.05	0.08	0.16	0.40	0.32	-
1063	(1*S*,3*S*)-(*E*)-4-Carene	-	-	-	-	0.03	-	-
1072	*γ*-Terpinene	0.12	0.04	0.06	0.11	0.32	0.38	-
1092	Terpinolene	0.65	0.14	0.21	0.44	1.41	3.70	-
1109	(+)-Verbenone	-	-	-	-	-	0.04	-
1114	1,5,8-*p*-Menthatriene	-	-	-	-	-	0.15	-
1158	Camphene hydrate	0.15	0.01	0.02	0.03	0.06	0.79	0.22
1213	3,6,6-Trimethylnorpinan-2-one	-	-	-	-	-	0.08	-
1250	Carvotanacetone	-	-	-	-	-	0.06	-
Oxygenated monoterpenes								
982	(1*S*,2*S*,3*R*,5*S*)-(+)-2,3-Pinanediol	-	-	-	-	0.04	-	-
1027	1,4-Cineole	-	0.02	0.03	0.06	0.14	0.09	-
1049	1,8-Cineole	-	0.01	0.02	0.04	0.09	0.10	-
1083	*ρ*-Menth-1-ene-3*β*,7-diol	0.15	0.04	0.06	0.12	0.36	1.10	-
1101	*α*-Pinene oxide	-	0.03	0.02	-	-	-	-
1121	Fenchol	0.77	0.05	0.12	0.25	0.61	5.36	0.72
1128	*α*-Campholenic aldehyde	0.23	0.04	0.05	0.09	0.18	1.28	-
1142	(−)-(*E*)-Pinocarveol	0.71	0.03	0.08	0.15	0.28	1.92	0.18
1149	(+)-Camphor	0.25	0.03	0.04	0.06	0.12	1.33	0.54
1152	*α*-Phellandren-8-ol	0.25	0.01	0.01	0.02	0.02	0.06	-
1163	(*E*)-Pinocamphone	0.10	0.02	0.04	0.07	0.12	1.26	0.50
1165	Pinocarvone	0.13	-	-	0.01	0.01	-	-
1166	Isoborneol	-	-	-	-	-	0.14	-
1180	Isopinocamphone	0.31	0.01	0.01	0.02	0.02	0.30	-
1183	Terpinen-4-ol	1.55	0.07	0.12	0.20	0.32	5.51	3.17
1190	*ρ*-Cymen-8-ol	0.28	-	0.02	0.02	0.04	0.74	0.60
1200	(+)-*α*-Terpineol	7.07	0.23	0.40	0.66	0.84	16.69	16.98
1202	(−)-Myrtenol	0.81	0.02	0.03	0.06	0.10	-	-
1202	Estragole	-	-	-	-	-	1.04	0.47
1212	(−)-Verbenone	0.30	0.01	0.02	0.02	0.02	-	-
1218	(−)-*β*-Fenchyl acetate	-	-	-	-	-	0.05	-
1220	(*R*)-Carveol	0.08	-	-	-	-	0.19	0.27
1228	Methyl thymol	-	-	-	-	-	0.14	-
1244	(+)-Carvone	-	-	-	-	-	0.06	0.16
1286	Bornyl acetate	1.26	0.03	0.04	0.06	0.07	1.63	3.51
1304	Perillyl alcohol	-	-	-	-	-	-	0.21
1343	Piperitenone	-	-	-	-	-	-	0.31
Sesquiterpene hydrocarbons								
1349	(−)-*α*-Cubebene	-	-	-	-	-	0.06	-
1354	*α*-Longipinene	1.09	0.01	0.01	0.02	0.02	0.60	2.24
1373	(+)-Cyclosativene	0.18	-	-	-	-	0.10	0.64
1379	*α*-Copaene	1.34	0.01	0.01	0.02	0.02	0.55	2.35
1396	(+)-Sativene	0.22	-	-	-	-	0.08	0.73
1405	(+)-Aromadendrene	-	-	-	-	-	-	0.15
1406	(−)-*α*-Gurjunene	-	-	-	-	-	-	0.24
1408	*γ*-Elemene	-	-	-	-	-	-	0.68
1415	Longifolene	8.77	0.04	0.05	0.07	0.06	2.61	27.87
1423	*β*-Caryophyllene	1.08	-	-	-	-	0.19	1.82
1436	*α*-Guaiene	-	-	-	-	-	-	0.29
1452	(*E*)-*β*-Famesene	0.24	-	-	-	-	-	-
1457	*α*-Humulene	0.26	-	-	-	-	-	0.78
1475	*γ*-Muurolene	-	-	-	-	-	-	0.21
1489	*α*-Ylangene	-	-	-	-	-	-	0.38
1491	*β*-Selinene	0.07	-	-	-	-	-	0.20
1495	(−)-*β*-Chamigrene	-	-	-	-	-	-	0.32
1498	*α*-Selinene	-	-	-	-	-	-	0.16
1501	*α*-Muurolene	0.51	-	-	-	-	0.05	1.77
1504	*β*-Himachalene	0.07	-	-	-	-	-	0.30
1512	*β*-Bisabolene	0.19	-	-	-	-	-	0.62
1514	(+)-Cuparene	-	-	-	-	-	-	0.16
1526	(+)-*δ*-Cadinene	0.47	-	-	-	-	-	1.46
1531	Calamenene	0.09	-	-	-	-	-	0.64
1559	*α*-Calacorene	-	-	-	-	-	-	0.14
1984	*β*-Elemene	-	-	-	-	-	-	1.10
Oxygenated sesquiterpenes								
1578	(*E*)-Nerolidol	0.24	-	-	-	-	-	0.30
1593	(−)-Caryophyllene oxide	0.24	-	-	-	-	-	-
1598	Viridiflorol	-	-	-	-	-	-	0.29
1609	Longiborneol	0.27	-	-	-	-	-	1.19
1639	Cubenol	0.16	-	-	-	-	-	0.63
1659	*δ*-Cadinol	0.08	-	-	-	-	-	0.31
1668	*α*-Cadinol	0.08	-	-	-	-	-	-
1694	*α*-Bisabolol	-	-	-	-	-	-	0.22
Diterpene hydrocarbons								
1951	Cembrene	8.36	-	-	-	-	-	0.28
2036	Manoyl oxide	0.07	-	-	-	-	-	-
2043	Pimara-8(14),15-diene	-	-	-	-	-	-	0.28
2151	Sclarene	-	-	-	-	-	-	0.15
2293	Isopimara-7,15-dien-3-one	0.12	-	-	-	-	-	0.22
Oxygenated diterpenes								
2083	Thunbergol	2.38	-	-	-	-	-	-
2092	Verticiol	-	-	-	-	-	-	0.32
2199	Sclareol	-	-	-	-	-	-	0.32
Triterpene hydrocarbons								
1855	Squalene	-	-	-	-	-	-	0.18
Monoterpene hydrocarbons		49.09	80.00	90.74	93.66	90.30	41.22	0.22
Oxygenated monoterpenes		14.27	0.65	1.11	1.91	3.39	38.99	27.62
Sesquiterpene hydrocarbons		14.58	0.06	0.07	0.11	0.10	4.25	45.27
Oxygenated sesquiterpenes		1.08	-	-	-	-	-	2.93
Diterpene hydrocarbons		8.56	-	-	-	-	-	0.93
Oxygenated diterpenes		2.38	-	-	-	-	-	0.63
Triterpene hydrocarbons		-	-	-	-	-	-	0.18
Others		10.03	19.29	8.08	4.32	6.21	15.54	22.20
Total identified		99.99	100.00	100.00	100.00	100.00	100.00	99.98

^a^ Kovats retention index was experimentally determined using DB-5MS column with a homologous series of C_8_–C_26_ alkanes. ^b^ Compounds are listed in order of their elution from the DB-5MS column.

## Data Availability

All data generated or analyzed during this study are included in this published article. Additional data are available from the corresponding author upon request.
